# On the mechanism of wogonin against acute monocytic leukemia using network pharmacology and experimental validation

**DOI:** 10.1038/s41598-024-60859-0

**Published:** 2024-05-02

**Authors:** Xixi Wang, Yanfei Wang, Jing Chen, Qinyao Wang, Zhongjian Liu, Yijie Yin, Tonghua Yang, Tao Shen, Yalian Sa

**Affiliations:** 1 Center for Clinical Medicine Research, The First People’s Hospital of Yunnan Province (Affiliated Hospital of Kunming University of Science and Technology), Kunming, 650032 China; 2 Medical School, Kunming University of Science and Technology, Kunming, 650500 China; 3 Department of Hematology, The First People’s Hospital of Yunnan Province, Kunming, 650032 China; 4 Department of Respiratory and Critical Care Medicine, The First People’s Hospital of Yunnan Province (Affiliated Hospital of Kunming University of Science and Technology), Kunming, 650032 China

**Keywords:** Cancer, Cell biology, Computational biology and bioinformatics, Drug discovery

## Abstract

Wogonin is a natural flavone compound from the plant Scutellaria baicalensis, which has a variety of pharmacological activities such as anti-cancer, anti-virus, anti-inflammatory, and immune regulation. However, the potential mechanism of wogonin remains unknown. This study was to confirm the molecular mechanism of wogonin for acute monocytic leukemia treatment, known as AML-M5. The potential action targets between wogonin and acute monocytic leukemia were predicted from databases. The compound-target-pathway network and protein-protein interaction network (PPI) were constructed. The enrichment analysis of related targets and molecular docking were performed. The network pharmacological results of wogonin for AML-M5 treatment were verified using the THP-1 cell line. 71 target genes of wogonin associated with AML-M5 were found. The key genes TP53, SRC, AKT1, RELA, HSP90AA1, JUN, PIK3R1, and CCND1 were preliminarily found to be the potential central targets of wogonin for AML-M5 treatment. The PPI network analysis, GO analysis and KEGG pathway enrichment analysis demonstrated that the PI3K/AKT signaling pathway was the significant pathway in the wogonin for AML-M5 treatment. The antiproliferative effects of wogonin on THP-1 cells of AML-M5 presented a dose-dependent and time-dependent manner, inducing apoptosis, blocking the cell cycle at the G2/M phase, decreasing the expressions of CCND1, CDK2, and CyclinA2 mRNA, as well as AKT and p-AKT proteins. The mechanisms of wogonin on AML-M5 treatment may be associated with inhibiting cell proliferation and regulating the cell cycle via the PI3K/AKT signaling pathway.

## Introduction

Acute monocytic leukemia is a subtype (AML-M5) of acute myeloid leukemia (AML) characterized by predominantly monoblasts and promonocytes, prone to increasing WBC count, hyperlipidemia, abnormal blood coagulation, and extramedullary infiltration^1–3^. Despite the great progress that has been made in treating AML-M5 with chemotherapy and biological target therapy, the 5-year survival rate (the 5y-SR) still has not improved significantly^4,5^. Thus, the unmet need for safer and more effective treatment strategies for AML-M5 is in anticipation.

Traditional Chinese medicine (TCM) is a commonly used alternative medicine therapy with multi-targets, multi-approach, cost-effective characteristics, and relatively few adverse reactions^5^. Of them, wogonin is a natural flavone compound from the plant Scutellaria baicalensis, which has a variety of pharmacological activities such as anti-cancer, anti-virus, anti-inflammatory, and immune regulation^6,7^.

Wogonin has been reported to exert immense therapeutic potential against cancer cells in various cancer types, such as bladder cancer, breast cancer, cholangiocarcinoma, cervical cancer, colorectal cancer, leukemia, multiple myeloma, and so on^6^. Particularly, wogonin has been demonstrated to reduce the production of NO and PGE2 and the expression of IL-1α, IL-1β, IL-6, GM-CSF, MCP-1, M-CSF, MIP-1α, MIP-1β, MIP-2, and COX-2 in macrophages and also to inhibit the infiltration of macrophages and neutrophils in tissue^8^. We speculate that wogonin may be a more promising candidate adjuvant therapeutic for the treatment of AML-M5. Himeji et al reported that wogonin has great potential effects on the inducing cycle arrest at the G2 /M phase and apoptosis of THP-1 cell line of AML-M5. And wogonin was found to be the most potent anti-cancer flavonoid compared to baicalein, baicalin, and wogonoside, which were all from Scutellaria baicalensis^9^. However, the mechanism underlying the therapeutic effects of wogonin in AML-M5 has not been fully clarified.

Network pharmacology combines systems biology, omics, and computational biology to analyze the mechanism of drug action with previous information^10^. Network pharmacology analyzes the correlation between action targets of drug components and diseases from a systematic and comprehensive perspective, to provide predictive information to illustrate the action mechanism of drugs^11^. Molecular docking technology can explore the binding pattern of small molecular compounds to targets and find out the compounds with the best affinity to targets^12^.

**Figure 1:**
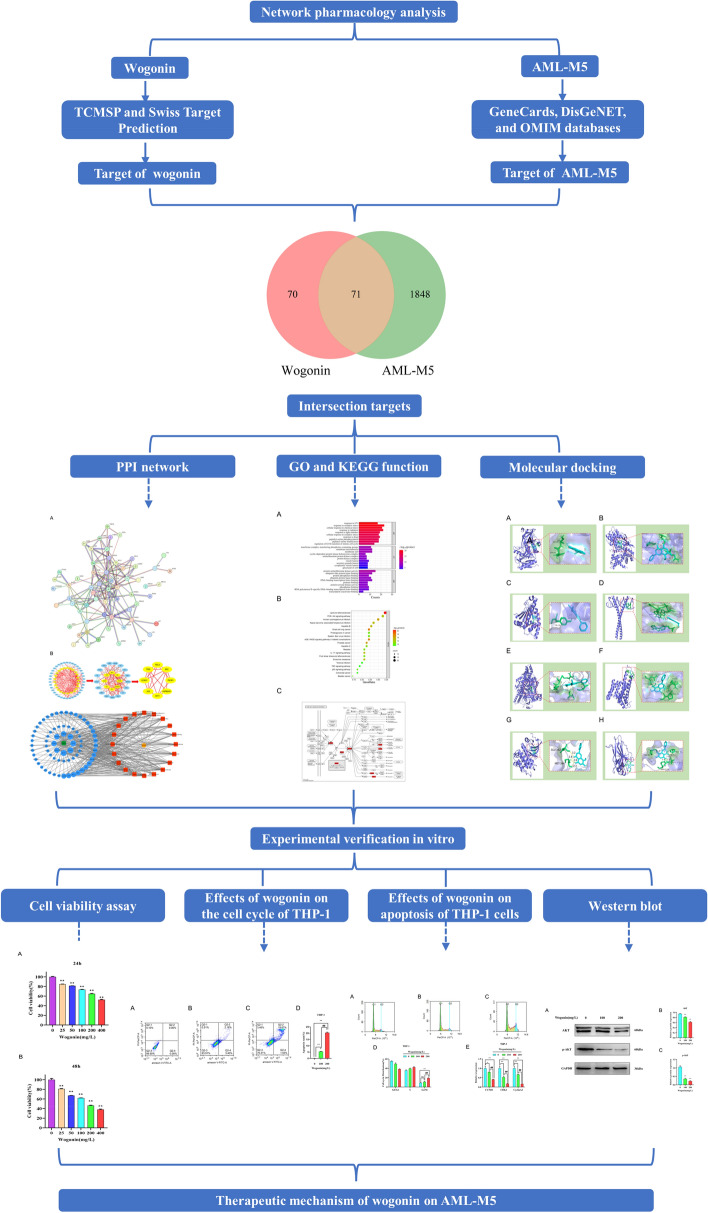
The schematic diagram of network pharmacology and molecular docking as well as experimental verification was used in this study.

In this study, we predicted the target of wogonin against AML-M5 using network pharmacology, bioinformatics, and molecular docking technology, and explored the mechanism of wogonin against AML-M5. For further validation, experiments in vitro were conducted to verify the pharmacodynamic effects of wogonin. Our findings may provide a theoretical basis for the clinical application of wogonin in treating AML-M5. The workflow diagram is shown in Fig. 1.

## Results

### Network pharmacology

#### Collection of potential targets

**Figure 2:**
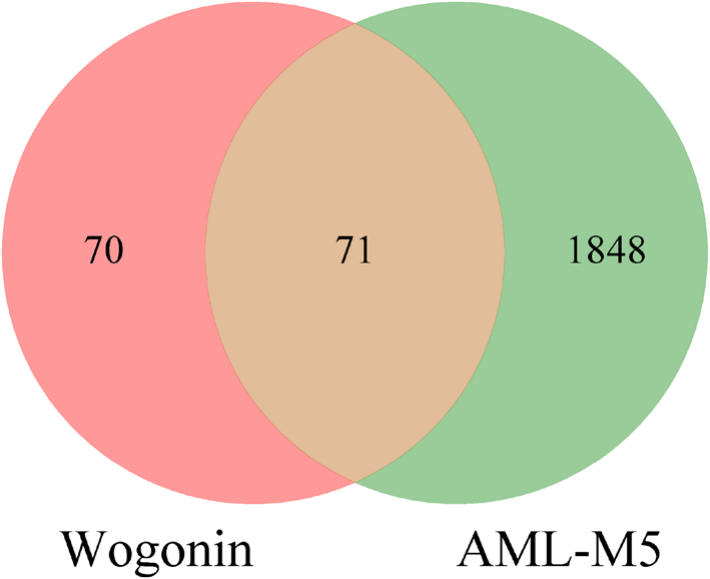
Venn diagram of the targets of wogonin in AML-M5.

A total of 141 potential targets for wogonin were retrieved from the TCMSP database and Swiss database. 1918 targets related to AML-M5 were found in GeneCards, OMIM, and DisGeNET. 71 genes were enriched after intersecting the targets of wogonin with AML-related targets (Fig. 2).

#### PPI network analysis

**Figure 3:**
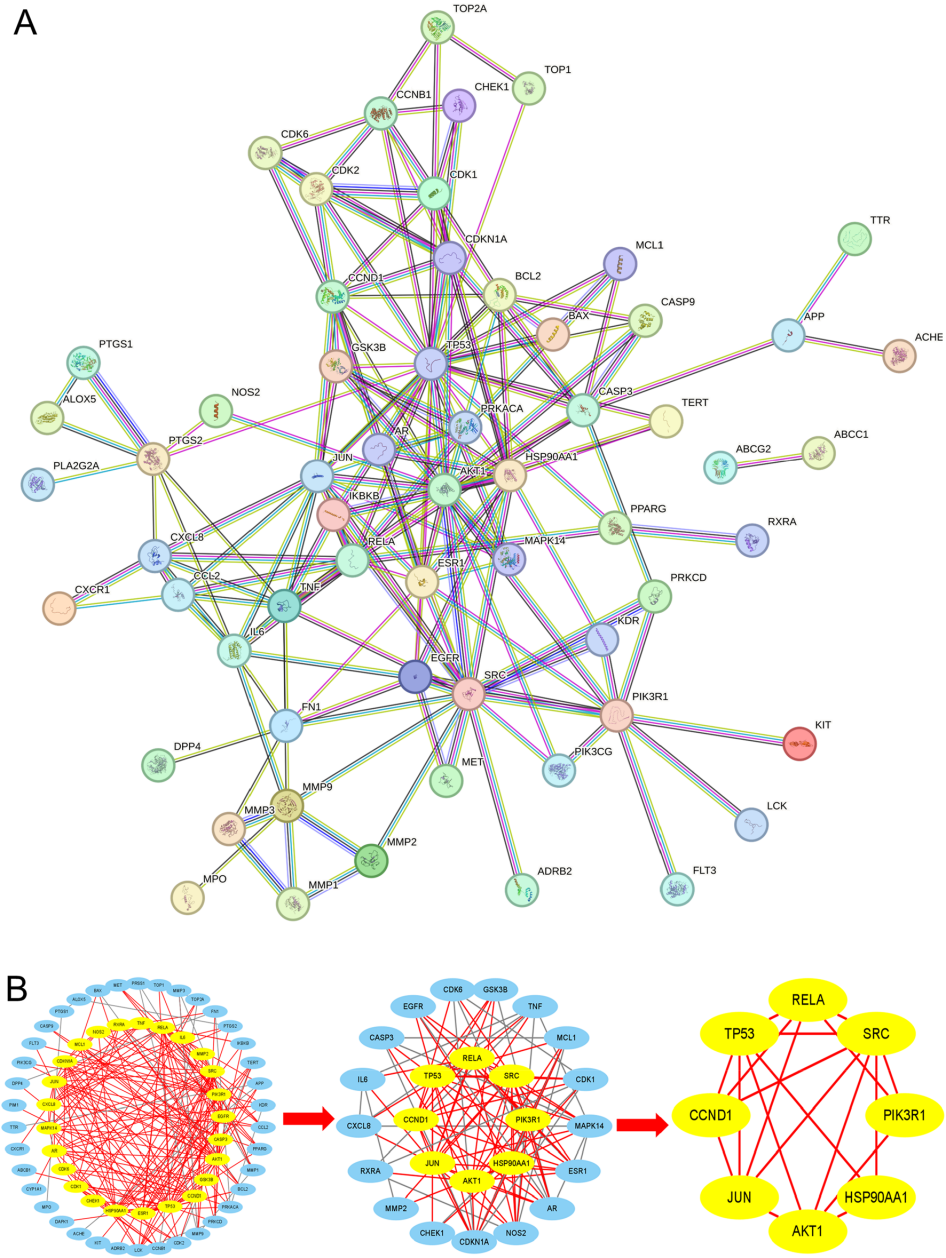
PPI network of potential target on AML-M5. (**A**) the construction of a protein interaction network of AML-M5 target genes induced by wogonin; (**B**) Screening of core targets of wogonin in the treatment of AML-M5.

To analyze the protein-protein interaction network, 71 potential genes were imported into the String platform to construct the PPI network (Fig. 3A). The network includes 71 protein nodes and 220 edges with an average node degree value of 602 and a clustering coefficient of 0.512. Then topology analysis was performed in Cytoscape 3.8.0 software using the plugin Cyto NCA, and median values of betweenness centrality (BC), closeness centrality (CC), and centrality (DC) were used as limiting conditions for screening, and all genes with indicators greater than the median were selected for subsequent analysis, and after two screenings, eight core targets were finally obtained, which were TP53, SRC, AKT1, RELA, HSP90AA1, JUN, PIK3R1, and CCND1(Fig. 3B, Table 1).

**Table 1:** Eight core target genes of AML-M5 were treated with wogonin.

Gene name	Protein name	BC	CC	DC
TP53	Cell tumor antigen P53	1038.733	0.569	26
SRC	Tyrosine-protein kinase SRC	627.329	0.559	23
AKT1	RAC-alpha serine/threonine-protein kinase	339.222	0.525	20
RELA	Transcription factor p65	464.514	0.534	18
HSP90AA1	Heat shock protein hsp90	263.780	0.492	18
JUN	Transcription factor AP-1	189.815	0.512	16
PIK3R1	PI3-kinase p85-alpha subunit	260.092	0.470	16
CCND1	G1/S-specific cyclin-D1	189.563	0.484	13

#### GO and KEGG pathway analysis

**Figure 4:**
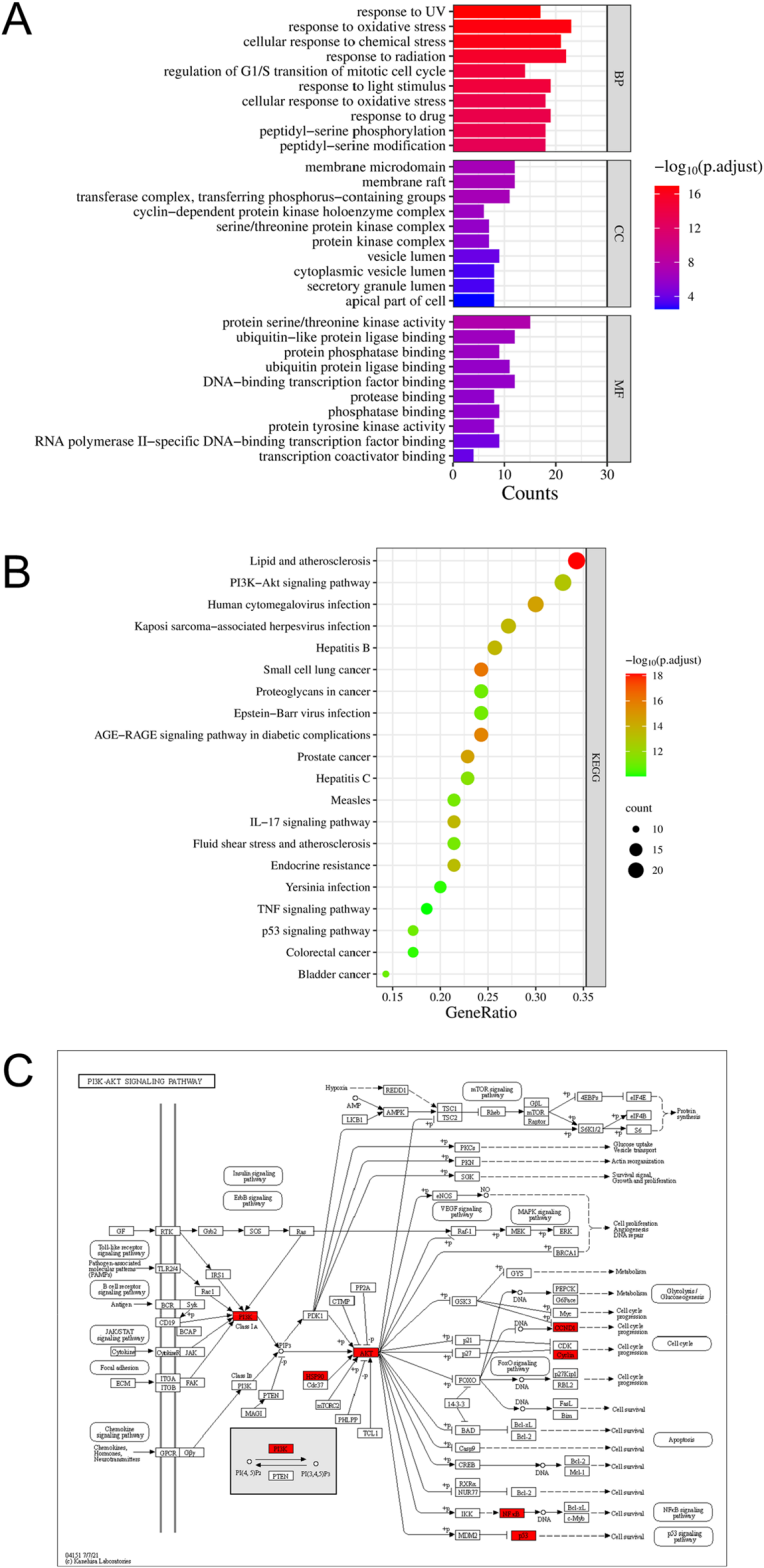
GO and KEGG pathway analysis. (**A**) The top 10 significance of enriched GO terms analysis of therapy target genes of wogonin on AML-M5 (*P* < 0.01). (**B**) Top 20 significance of enriched KEGG pathways and analysis of PI3K-AKT signal path in KEGG database (*P* < 0.01). (**C**) The font marked in red represents the target in the PI3K-AKT signal pathway closely related to treating AML-M5 with wogonin.

To gain more insights into the cellular function of wogonin in AML-M5, we performed a GO term enrichment analysis. The results indicated that the common protein targets have multiple biological functions. The top 10 significantly enriched terms in each category are shown in Fig. 4A. Major terms in the BP category involved oxidative stress, radiation, and chemical stress. Major CC terms included membrane raft, membrane microstructure domain, and transfer of phosphorus-containing groups. Major MF terms covered serine/threonine kinase activity, ubiquitin-like protein ligase binding, and DNA binding transcription factor binding. The KEGG pathway enrichment analysis results showed that wogonin in the treatment of AML-M5 mainly focused on the PI3K-Akt signaling pathway, lipid, and atherosclerosis, human cytomegalovirus infection, Kaposi's sarcoma-related herpesvirus infection, and hepatitis B (Fig. 4B). Furthermore, we analyze of PI3K-AKT signal pathway in the KEGG database. The font marked in red represented the key targets closely with the AML-M5 in the treatment of wogonin by the PI3K-AKT signal pathway (Fig. 4C).

#### Construction of compound-target-pathway network diagram

**Figure 5:**
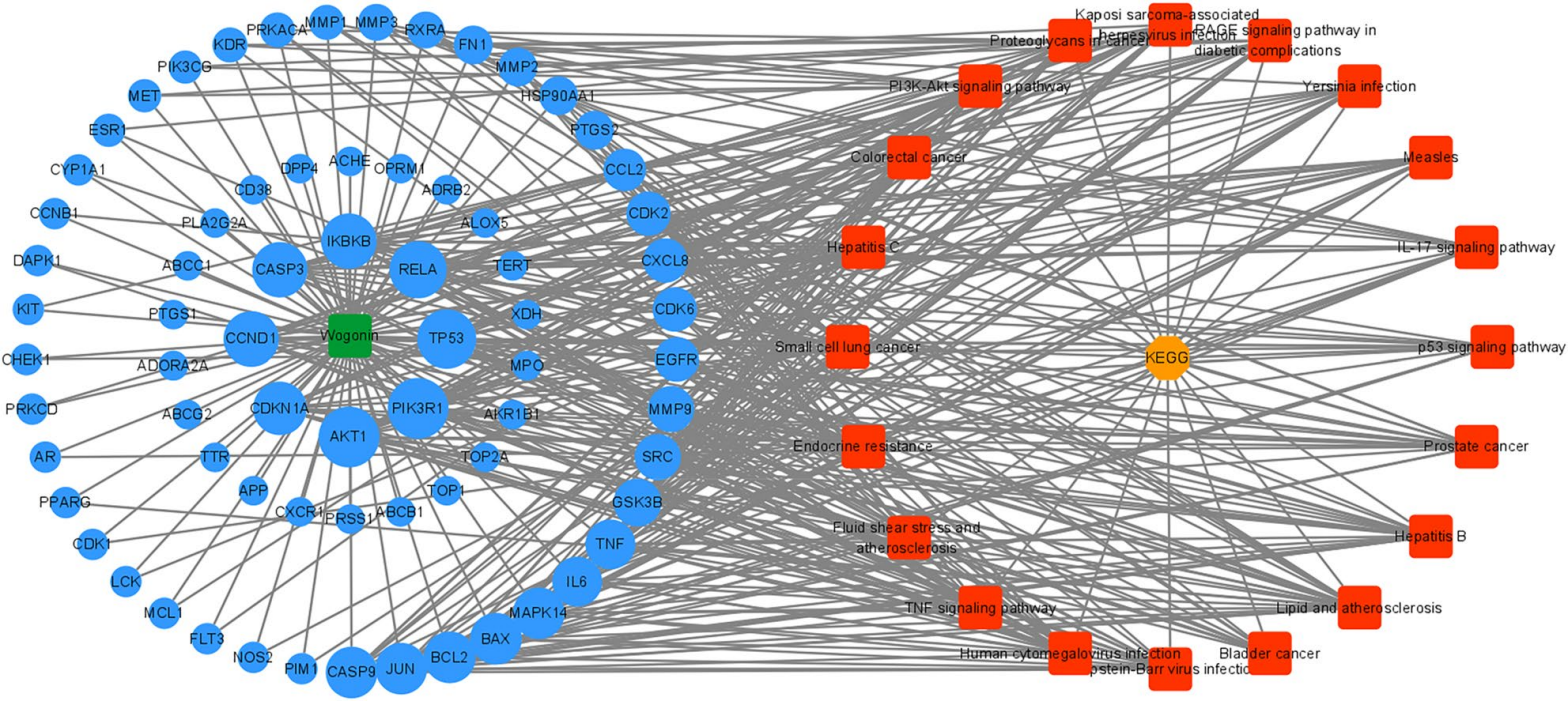
Compound-target-pathway networks of wogonin in AML-M5. The blue circles correspond to targets, node size is proportional to their degree, and red rectangles represent pathways.

A compound-target-pathway network diagram was constructed by combining the target of wogonin in treating AML-M5 with the related pathways obtained by KEGG enrichment analysis with Cytoscape 3.8.0 software (Fig. 5). The network consists of 93 nodes and 417 edges. Among them, the AKT1 degree value is 18 at the highest. Therefore, it is speculated that AKT1 is the key target of wogonin against AML-M5.

#### Molecular docking

**Figure 6:**
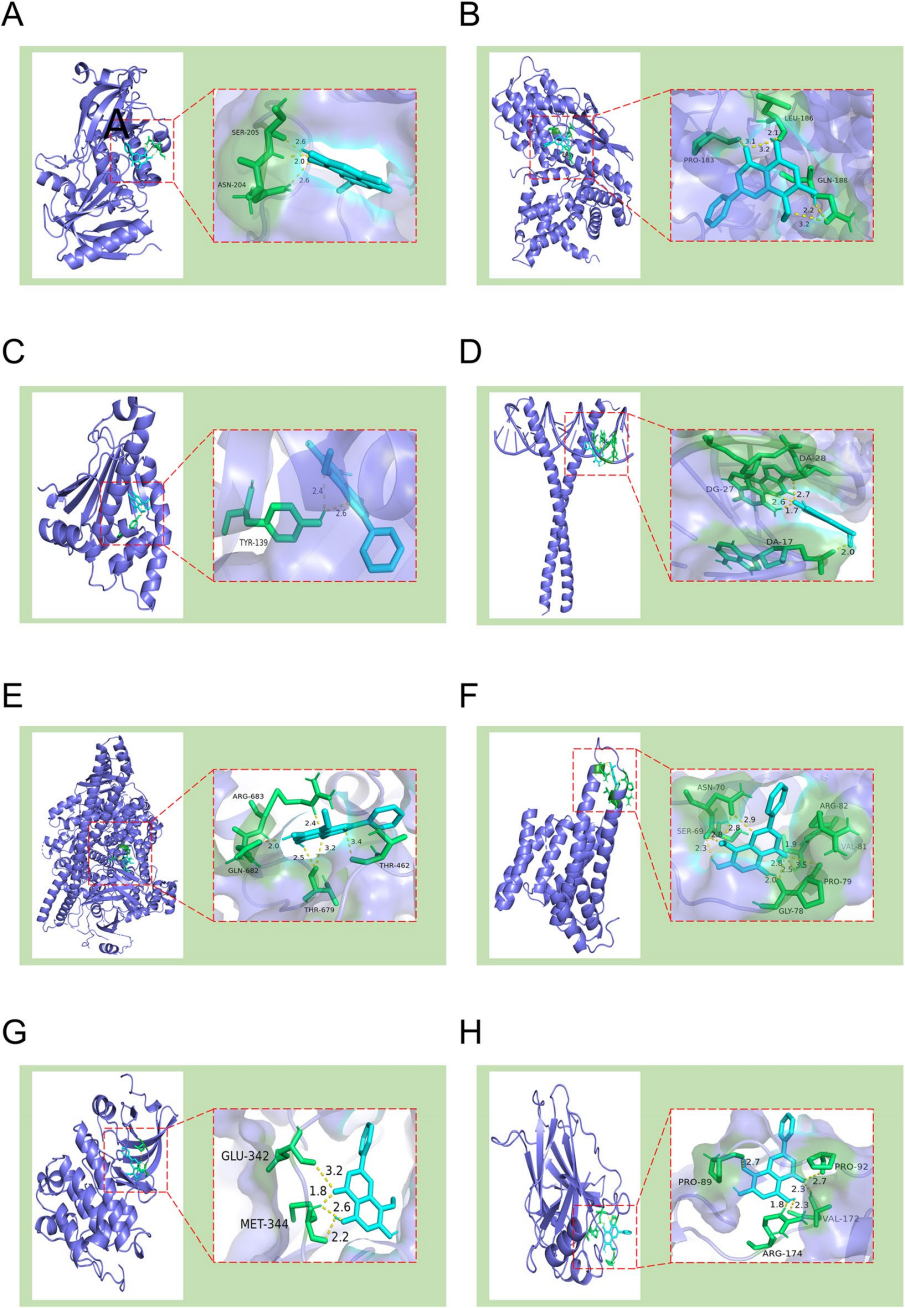
Docking diagram of wogonin and core targets. (**A**) AKT1. (**B**) CCND1. (**C**) HSP90AA1. (**D**) JUN. (**E**) PIK3R1. (**F**) RELA. (**G**) SRC. (**H**) TP53.

The binding energy < -5.0 kcal/mol indicated good binding activity, and the binding energy < -7.0 kcal/mol indicated strong binding activity^13^. The results of molecular docking showed that the binding energies of the above-mentioned target proteins and compounds were less than -5.0 kcal/mol (Table 2, Fig. 6), which indicated that the above-mentioned eight core targets had good binding activities with wogonin. The binding energy of wogonin and AKT1 was -7.34 kcal/mol, and three hydrogen bonds were formed between ASN-204 and SER-205 residues of the target protein.

**Table 2:** The binding energy in molecular docking between wogonin and core targets.

Target protein name	the Protein Data Bank (PDB) ID	Binding energy (kcal/mol)
HSP90AA1	7LSZ	− 7.75
PIK3R1	4WAF	− 7.48
AKT1	7NH5	− 7.34
CCND1	2W96	− 7.01
SRC	7NG7	− 6.71
TP53	6SL6	− 6.53
RELA	1NF1	− 6.00
JUN	5T01	− 5.86

### Experimental verification in vitro

#### Wogonin inhibits the growth of THP-1 cells

**Figure 7:**
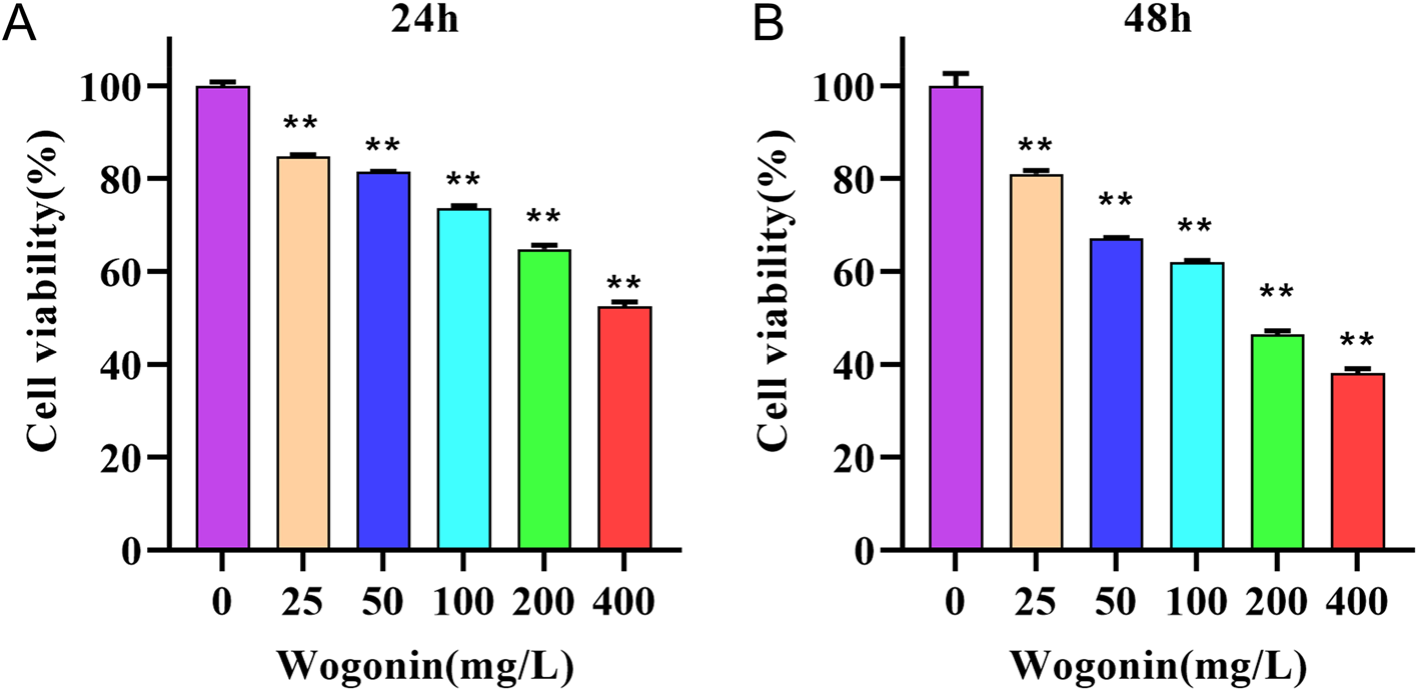
Wogonin inhibits the proliferation of THP-1 cells. (**A**,**B**) THP-1 cells were treated with different concentrations of wogonin for 24 h and 48 h. **P < 0.01 compared with the control group. All data were analyzed as mean ± SD (*n* = 3). One-way ANOVA followed by the SNK method.

To investigate the dose effects of wogonin, we treated THP-1 cells with various concentrations of wogonin for 24 h and 48 h, then CCK-8 assay was employed to test the cell viability. As shown in Fig. 7, wogonin dose-dependently reduced the viability of THP-1 cells with IC50 values of 492.2 mg/L at 24 h and 179.6 mg/L at 48 h, respectively. THP-1 cells treated with wogonin with 100 mg/L and 200 mg/L for 48 h presented with significantly reduced cell numbers compared with the control group. Therefore, we chose the concentration of wogonin with 100 mg/L and 200 mg/L at 48 h for further study.

#### Effects of wogonin on apoptosis of THP-1 cells

**Figure 8:**
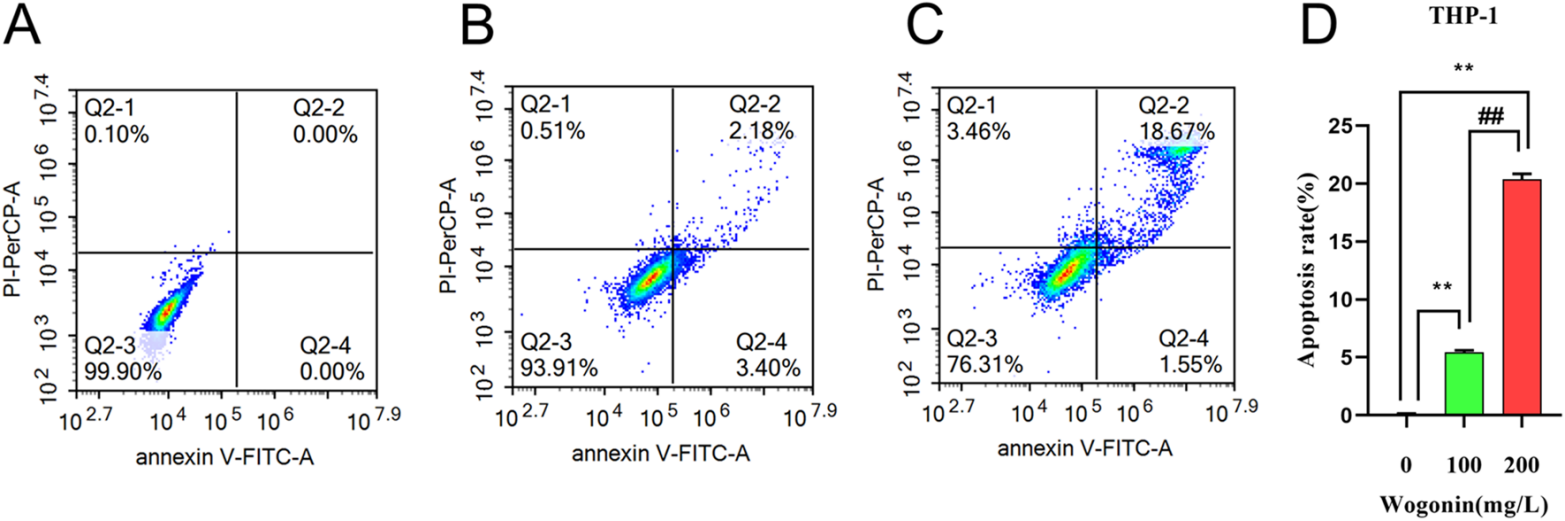
Apoptosis analysis was detected by flow cytometry. (**A**–**C**) THP-1 cells were treated with wogonin at indicated concentrations for 48 h. (**D**) Quantitative analysis of the percentage of apoptotic cells. **P < 0.01 compared with the control group. ##*P* < 0.01 compared with 100 mg/L wogonin treatment group. All data were analyzed as mean ± SD (*n* = 3). Student's *t*-test was used for statistical analyses of the 100 mg/L and 200 mg/L wogonin treatment groups, One-way ANOVA followed by the SNK method was used for statistical analyses of the control group and the 100 mg/L and 200 mg/L wogonin treatment group.

To characterize the effect of wogonin on cell death of THP-1 cells, we examined the apoptosis rate of cells treated with wogonin by annexin V-FITC and PI staining by flow cytometry analysis. As shown in Fig. 8, flow cytometry analysis showed that wogonin promoted cell apoptosis in a dose-dependent manner. After being treated with wogonin for 48 h, 5.58% ± 0.14%, and 20.22% ± 0.38% apoptotic cells were found in THP-1 cells treated with 100 mg/L and 200 mg/L, which were significantly higher than that in the control group (0.00% ± 0.06%). These data suggest that wogonin induces cell apoptosis in the dose-effect relationship.

#### Effects of wogonin on the cell cycle of THP-1 cells

**Figure 9:**
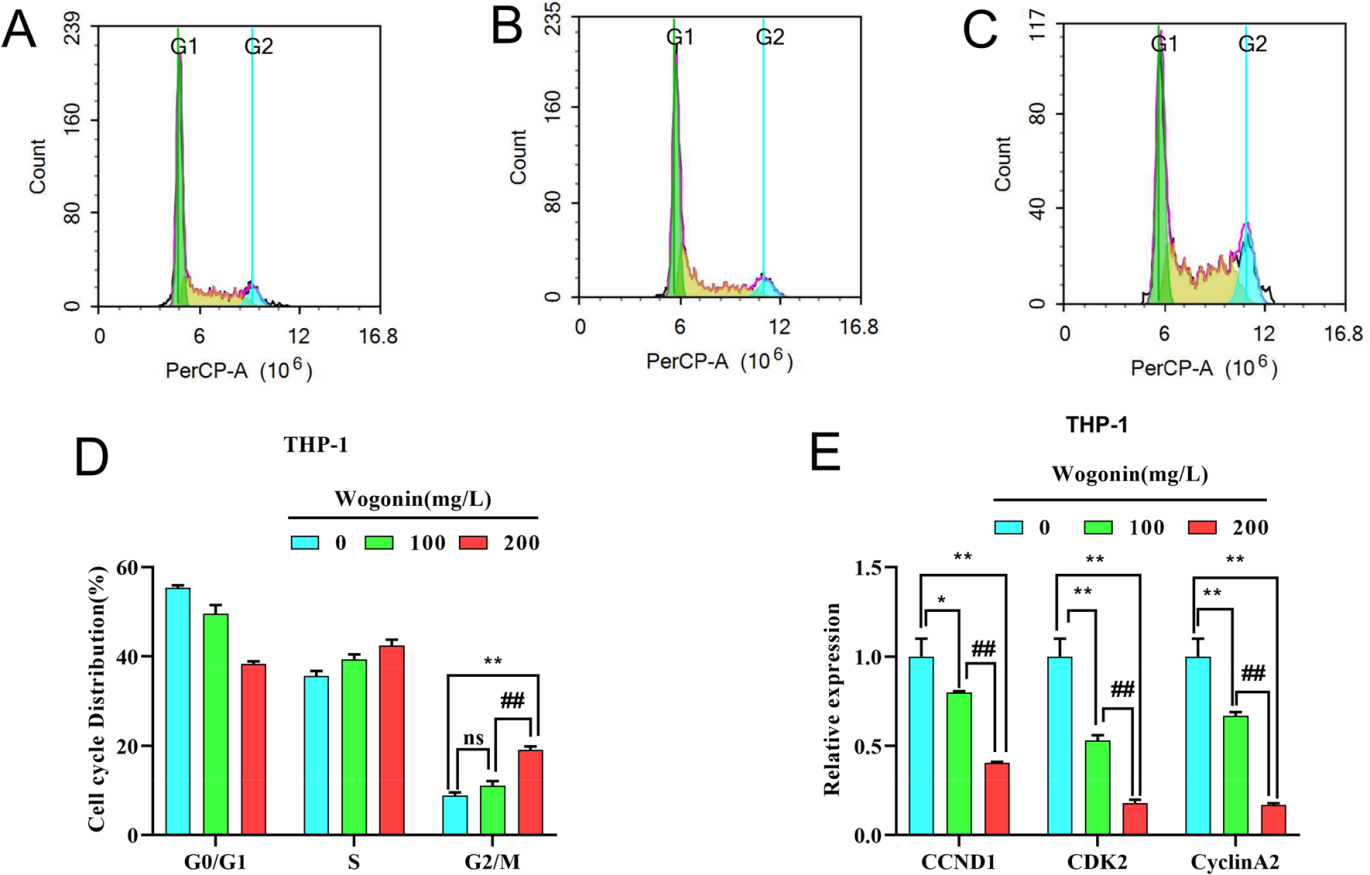
The cell cycle of THP-1 cells by flow cytometry analysis. (**A**–**C**) The distribution of THP-1 cells treated with wogonin was shown in different phases of the cell cycle. (**D**) Quantitative analysis of the cell cycle phase. **P < 0.01 compared with the control group. ^##^*P* < 0.01 compared with 100 mg/L wogonin treatment group (*n* = 3). (**E**) Validation by qRT-PCR analysis of altered expression of genes related to cell cycle that were selected based on network pharmacology. **P < 0.01, *P < 0.05 compared with the control group. ^##^*P* < 0.01 compared with 100 mg/L wogonin treatment group. All data were analyzed as mean ± SD (*n* = 3). Student's *t*-test was used for statistical analyses of the 100 mg/L and 200 mg/L wogonin treatment groups, One-way ANOVA followed by the SNK method was used for statistical analyses of the control group and the 100 mg/L and 200 mg/L wogonin treatment group.

The results of GO and KEGG analysis suggested that wogonin could induce cell cycle arrest of THP-1 cells. The results of flow cytometry showed that with the increase of wogonin concentration, the percentage of cells also accumulated in the G2/M phase from 9.63% ± 0.52%, 10.02% ± 0.85% to 18.4% ± 0.66% at 0 mg/L, 100 mg/L and 200 mg/L, respectively (Fig. 9A–D). qRT-PCR results showed that wogonin could inhibit the mRNA expression of CCND1, CDK2, and CyclinA2, compared with the control group (Fig. 9E). These results demonstrate that wogonin inhibits the growth of THP-1 cells probably via inducing G2/M phase arrest.

#### Wogonin regulated the PI3K-AKT signaling pathway in THP-1 Cells

**Figure 10:**
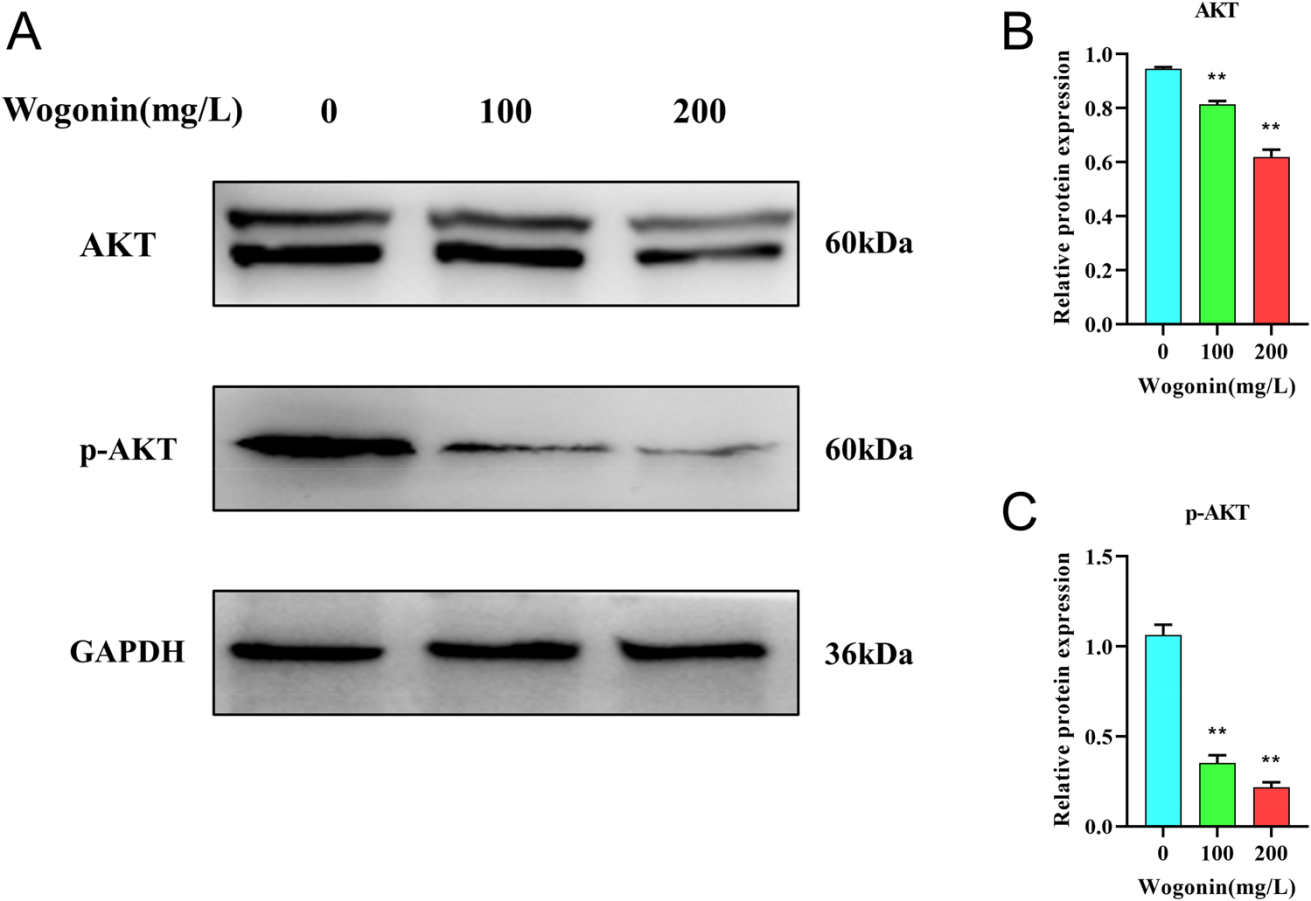
The protein expression levels of AKT and p-AKT in THP-1 cells treated by wogonin. (**A**) The protein expression levels of AKT and p-AKT were measured by western blot assay. (**B**,**C**) Quantitative analysis of protein expression levels of AKT and p-AKT. **P < 0.01 compared with the control group. All data were analyzed as mean ± SD (*n* = 3). One-way ANOVA followed by the SNK method.

According to network pharmacology analysis, the PI3K/AKT pathway is the key signaling axis associated with the effect of wogonin against AML-M5. Thus, we analyzed the levels of protein in this pathway by western blotting. As shown in Fig. 10, AKT, p-AKT showed a dose-dependent decrease after wogonin treatment compared to the control group.

## Discussion

AML-M5 is an incurable hematological malignancy characterized by abnormal proliferation, apoptosis repression, and differentiation blockage of hematopoietic stem/progenitor cells (HSCs/HPCs)^14^. A large body of evidence indicates that traditional Chinese medicine including wogonin plays an important role in killing cancer cells including leukemia cells^6^. Here, by network pharmacology, molecular docking, and experimental validation in vitro, we demonstrated the molecular mechanism of wogonin for AML-M5 treatment. Our data shows that the PI3K/AKT pathway contributes to inhibiting proliferation, inducing apoptosis, and blocking the cell cycle phase of the THP-1 cells line derived from AML-M5 treatment by wogonin, which is associated with the expression decreasing of CCND1/CDK2/CyclinA2 mRNA and AKT protein.

TCM has been used to treat numerous kinds of diseases for more than 2000 years in China, but the mechanism of action is challenging due to the diverse ingredients, their complex interaction with the human body, and their potential toxicity^5,15^. Although generally assumed that a synergism of all ingredients will bring about the maximum therapeutic efficacy, a traditional Chinese pharmaceutical monomer is the promising way^16^. Wogonin, a flavonoid compound extracted from the roots of Scutellaria baicalensis, has been found to possess a variety of pharmacological activities, including anti-AML effects^5,6^. Wogonin demonstrated anti-leukemia effects in K562, KU-812, and primary chronic myeloid leukemia (CML) cells through modulating P-TEFb activity in vitro as well as in vivo^17^. Moreover, wogonin inhibited the viability of HL-60 cells, which are human promyelocytic leukemia cells, through caspase-dependent and mitochondrial-dependent apoptosis. Additionally, wogonin induced the expression of key members of the endoplasmic reticulum (ER) stress pathway, such as CHOP, GRP94, and GRP78^18^. It also activated ER stress transducers such as IRE1α, PERK-eIF2α, and ATF6 in HL-60 cells by inhibiting the PI3K-AKT signaling pathway. Dürr C et al. reported that wogonin inhibited the expression level of tumor necrosis factor (TNF) receptor-1 in chronic lymphocytic leukemia associated with the NF-κB pathway^19^. Furthermore, wogonin could significantly reverse the resistance of K562 and KU812 cells to imatinib (IM) by controlling the TGF-β/Smad4/Id3 pathway and decreasing the expression of CXCR4 and CXCR7^20^. Taken together, these data suggested that wogonin exhibited anti-leukemia effects. However, the pharmacological mechanism of wogonin against AML-M5 is still uncompleted.

Using network pharmacology in this work, the 141 potential therapeutic targets of wogonin on AML-M5 were obtained, and the PPI Network demonstrated the relationships between wogonin and AML-M5 represented by 71 nodes and linked with the edges involved eight core candidate target genes as following: HSP90AA1, PIK3R1, AKT1, CCND1, SRC, TP53, RELA and JUN. Furthermore, the results of GO and KEGG pathway analysis revealed AKT signaling pathway was critical for wogonin-induced apoptosis and inhibited the proliferation of THP-1 cells. Molecular docking and dynamics simulation further demonstrated that wogonin bound best to AKT1. In vitro, we validated that wogonin significantly inhibited the growth of THP-1 cell lines of AML-M5 and induced apoptosis in a dose- and time-dependent manner. Moreover, wogonin treatment markedly induced G2/M phase cell cycle arrest, which was accompanied by downregulation of CCND1, CDK2, and cyclin A2. The results from in vitro experiments were consistent with those from network pharmacology. Therefore, our finding results suggested that AKT may be a new target for wogonin in the treatment of AML-M5. The key targets are discussed in detail below.

CCND1 is located on chromosome 11q13 and encodes cyclin D1 protein associated with cell cycle progression, and is frequently overexpressed in many cancer types, including leukemia^21^. CircPLXNB2 was highly expressed in AML patients and cells and modulated tumor progression by regulating the circPLXNB2/miR-654-3p/CCND1 axis^22^. CCND1 overexpression decreased the sensitivity of AML cells to Ara-C^23^. CCND1-BCL2 gene network corresponds to a cell cycle arrest in the G2/M phase and cell apoptosis of Dami cells of human acute megakaryocytic leukemia treated by amifostine^24^. The mixture of GX guttiferone E and xanthohumol-derived compound 2 induced apoptosis and arrested the cell cycle in the HEL leukemic cell line by reducing the expression of anti-apoptotic protein Bcl-2 and cell cycle-specific cyclin D1 and by enhancing the pro-apoptotic protein Bax^25^. CDK2 is a key regulator of the eukaryotic cell cycle significantly over-activated in many cancers. Inhibition of CDK2 reduces proliferation in leukemic cells invading the spleen in MYC/BCL-XL mice^26^. Benzo chromene suppressed cell growth in HL-60 by the induction of cell cycle arrest at the G1/S phase by regulating the expression of CDK-2/CyclinD1, triggering cell apoptosis by activating both the extrinsic (Fas/Caspase 8) and intrinsic (Bcl-2/Caspase 3) apoptosis pathways^27^. The anti-proliferative, cell cycle regulation and apoptosis-inducing capacity of doxorubicin (DOX) in THP-1 leukemia cells were with the downregulation of CDK2^28^. Notopterol could induce apoptosis, differentiation, and G0/G1 arrest in human AML HL-60 cells which may be related to the regulation of cell-cycle-related proteins p53, CDK2, CDK4, Cyclin D1, Cyclin E, and surviving^29^. Cyclin A2 expression occurs in the S and G2 phases of the cell cycle regulating both spatial and temporal phosphorylation of target proteins. The PLK4 inhibitor centrinone and the shRNA knockdown induced AML cell apoptosis by increasing the activation of Caspase-3/poly ADP-ribose polymerase (PARP), and caused the G2/M phase cell cycle arrest by decreasing the expression of cell cycle-related proteins such as Cyclin A2, Cyclin B1, and Cyclin-dependent kinase 1 (CDK1)^30^. The cytotoxicity of vitamins K2 and K3 on human T lymphoblastoid leukemia cells, Jurkat T cells, and MOLT-4 cells seems to be related to apoptosis induction and cell cycle arrest by down-regulated the expressions of cyclin A2^31^. In agreement with these results, the expression of CCND1, CDK2, and cyclin A2 were notably reduced in THP-1 cells treated with wogonin, which may be associated with the cells accumulated in the G2/M phase. And these findings supported that wogonin selected inhibition of CDK9-overexpressing MV4-11 cell line of AML-M5 through caspase-dependent apoptosis reported by Wang and colleagues^32^.

PI3K/AKT has been extensively studied in normal and malignant cells, which are involved in many cellular processes including cell survival, proliferation and differentiation, etc^33^. There are three structurally active forms of Akt in mammalian cells named Akt1, Akt2, and Akt3 or PKB α, β, γ, respectively^34^. The signaling cascade is activated by a wide variety of extracellular stimuli, including receptor tyrosine kinases, various integrins, B and T cell receptors, and G-protein-coupled receptors (GPCRs)^35^. This activation leads to the relocation of Akt to the cytosol or the nucleus, and the expression of target genes^36^. Our results of western blotting showed the lowered activity of Akt as well as downregulated expression of p-Akt in the THP-1 cells treated by wogonin, which may related to inhibite proliferation, induce apoptosis, and result in G2/M arrest in THP-1 cells.

However, there were still limitations in the present study. All key targets screened by enrichment analysis requiring experimental validation at mRNA and protein in vitro. Moreover, Akt antagonists might be considered to treat THP-1 cells. Furthermore, only the THP-1 cell line was included in our study, the effects of wogonin on more AML-M5 cell lines would be considered. The last one but the best one, the mice in vivo model of AML-M5 should be analyzed. The Discussion should be succinct and must not contain subheadings.

In conclusion, the present study demonstrated that wogonin inhibited the proliferation of THP-1 cells in vitro. Mechanistically, wogonin treatment suppressed AKT activation, leading to cell cycle arrest and apoptosis. Therefore, our finding results suggest that AKT may be a target for wogonin in the treatment of AML-M5.

## Methods

### Network pharmacology

#### Screening of therapeutic targets of wogonin and AML-M5

We hypothesize that the targets of wogonin intersect with the targets of AML-M5 were potential therapeutic targets of wogonin on AML-M5. The targets of drug action were obtained through the TCMSP database^37^ (https://old.tcmsp-e.com/tcmsp.php) and the Swiss database^38,39^ (http://www.swisstargetprediction.ch/). Disease targets were obtained through the GeneCards^40^ (https://www.genecards.org/), DisGeNET^41^ (https://www.disgenet.org/), and OMIM databases^42^ (https://www.omim.org). They were then normalized by the UniProt database^43^ (http://www.uniprot.org/help/uniprotkb).

#### Screening of common targets for drug-diseases

The intersection of wogonin against AML-M5 was obtained through the Venny online website. The intersection targets were uploaded to the String platform^44^ (http://stringdb.org/) to construct protein-protein interaction (PPI) Networks with the organism selected of Homo sapiens sources, and the confidence level of protein interaction was set as *P* > 0.9. The interaction information of protein-protein as well as the core genes was analyzed by using the Cytoscape 3.8.0 software^45,46^ and the Cyto NCA plug-in. In the PPI network diagram, each node represents a protein, and the connection between nodes indicates an interaction between two proteins.

#### GO and KEGG enrichment analysis

The information on intersection targets was input to the DAVID platform^47,48^ (https://david.ncifcrf.gov/summary.jsp) for the gene ontology (GO) and the Kyoto Encyclopedia of Genes and Genomes (KEGG)^49–51^ pathway enrichment analyses. GO functional enrichment analyses were annotated into three terms: biological processes (BP), cell composition (CC), and molecular function (MF). KEGG pathway enrichment analysis is used to clarify the potential mechanism of wogonin for AML-M5. In this study, statistical significance was set at *P* value ≤ 0.05, and the bar charts and bubble diagrams were drawn using the micro-information visual cloud platform (http://www.bioinformatics.com.cn).

#### Construction of compound-target-pathway network diagram

Based on GO and KEGG data information, we identified and visualized the com-pound-target-pathway relationship between wogonin and AML-M5 achieved with Cytoscape 3.8.0 software.

#### Molecular docking

The main compounds of wogonin and key protein targets were analyzed by molecular docking using the AutoDockTools 1.5.7 software^52,53^. The 3D structures of wogonin were obtained from the TCMSP database. The 3D structures of key protein targets were obtained from the RCSB Protein Data Bank (RCSB PDB) database^54^ (https://www.rcsb.org/). The figures of the active binding site were generated with the PyMOL 2.2.0 software (https://pymol.org/2/).

### Experimental verification

#### Drug and reagents

The standard sample of wogonin was purchased from Guizhou Dida Technology Co., Ltd. Roswell Park Memorial Institute (RPMI) 1640, and fetal bovine serum (FBS) was purchased from Gibco Life Technologies Ltd. Cell Counting Kit-8 (CCK-8) kit, RIPA lysis buffer, PMSF, SDS-PAGE Sample Loading Buffer, and BCA protein quantitation kit were purchased from Beyotime Institute of Biotechnology. DMSO was purchased from Sig-ma-Aldrich Trading Co., Ltd (Shanghai, China). Annexin FITC/PI apoptosis detection kit and cell cycle detection kit were purchased from BD Bioscience. RNA extraction kit, Re-verse transcription kit, and SYBR Green Master Kit were purchased from TaKaRa Company (Dalian, China). Rabbit-derived primary antibody AKT1 and Rabbit-derived primary antibody p-AKT were purchased from Cell Signaling Technology, Inc. (Shanghai, China). Mouse-derived primary antibody GAPDH was purchased from Servicebio (Wuhan, China). Goat anti-rabbit IgG and Goat anti-mouse IgG were purchased from Protein Tech Group, Inc. (Wu Han, China). Tris-base and the rest of the reagents were provided by Solabio Life Sciences (Beijing, China).

#### Cell culturing

THP-1 cells were frozen for this clinical medical research center. THP-1 cells were cultured in RPMI-1640 medium supplemented with 10% fetal bovine serum (FBS) and 1% penicillin (pen) and streptomycin (strep) at 37 °C in an incubator containing 5% CO_2_.

#### Cell viability assay

The viability of the THP-1 cells was determined using a CCK-8 kit. THP-1 cells were inoculated in 96-well plates at a volume of 100 μl/well, then 10 μl medium containing various doses of wogonin (0, 25, 50, 100, 200, and 400 mg/L) was added and incubated for 24 h and 48 h. Subsequently, 10 μl/well CCK-8 solution was added and incubated for 2 h. The BioTek H1MF microplate reader (BioTek Instruments, SYNERGYH1MF) was used to measure the absorbency (OD) at 450 nm. The cell viability was calculated with the following equation: Cell viability (%) = [OD (treated) − OD (blank)]/[OD (control) − OD (blank)] × 100%. The IC50 of wogonin was counted by Graph Pad Prism 8.0 software. In the following experiment, we added 100 mg/L or 200 mg/L wogonin to the culture medium of THP-1 cells at 48 h.

#### Flow cytometry for Cell cycle and apoptosis analysis

Cell cycle analysis was performed to quantify the cellular DNA content. Cells were treated as described above. After incubation, THP-1 cells were harvested, washed with PBS, and fixed in 70% ethanol (minimum 24 h; 4 °C). Following ethanol fixation, the cells were washed in PBS and centrifuged at 300 × *g* for 5 min at 4 °C. Next, the cells were washed, suspended in PI staining solution, and incubated for 30 min at room temperature away from light. The stained cells were filtered and analyzed using flow cytometry (ACEA NovoCyte Penteon, Agilent Technology), and the percentage of cells in each cell phase was calculated.

Apoptosis was analyzed using an Annexin V-FITC/PI staining kit according to the manufacturer's instructions. THP-1 cells were centrifuged in the tube rinsed twice with pre-cold PBS buffer, then incubated with 5 µl PI and 5 µl FITC Annexin V in 100 µl 1×Binding Buffer at room temperature in the dark for 15 min. Next, THP-1 cells were rinsed with pre-cold PBS buffer, then the cells were suspended in 400 μl 1×Binding Buffer, and analyzed using a flow cytometer.

#### Quantitative real-time polymerase chain reaction (qRT-PCR) analysis for gene expression

Total RNA from THP-1 cells was extracted utilizing the TaKaRa Kit, 1 μg of which was applied for cDNA synthesis. qRT-PCR was followed using SYBR Green Master Mix with Roche LightCycler 480 real-time PCR system. The relative mRNA expression of tested genes was calculated with 2^−△△CT^, method using GAPDH as the housekeeper gene. Primers involved in our work are listed in Table 3.

**Table 3:** Primer sequences for the amplification of different genes by qRT-PCR.

Gene	Sequence (5′-3′)
GAPDH-F	GTCTCCTCTGACTTCAACAGCG
GAPDH-R	ACCACCCTGTTGCTGTAGCCAA
CCND1-F	TCTACACCGACAACTCCATCCG
CCND1-R	TCTGGCATTTTGGAGAGGAAGTG
CDK2-F	ATGGATGCCTCTGCTCTCACTG
CDK2-R	CCCGATGAGAATGGCAGAAAGC
CyclinA2-F	CTCTACACAGTCACGGGACAAAG
CyclinA2-R	CTGTGGTGCTTTGAGGTAGGTC

### Western blotting

THP-1 cells were inoculated in 6-well plates and incubated in a medium containing a variety of wogonin concentrations (0, 100, and 200 mg/L) for the indicated time point. Cell lysates were prepared with RIPA buffer supplemented with phosphatase inhibitor and protease inhibitor. Protein concentrations were quantified by the BCA protein assay kit. Protein samples were heated at 95 °C for 10min following diluted in 5×loading buffer. Then, 20 µg of each sample was fractionated on 10% SDS-PAGE gels and transferred to the PVDF membrane. The PVDF membranes were blocked with blocking solution for 1h and incubated with different primary antibodies (1:1000) overnight at 4 °C. Then, an HRP-conjugated secondary antibody (1:2000) was added at room temperature and the membrane was washed with 1×TBST. Proteins were visualized with enhanced chemiluminescence (ECL) detection reagents. Semi-quantitative analysis was performed by using Image J software. Target protein levels were normalized by the level of GAPDH.

### Statistical analysis

All statistical analyses were performed using SPSS 25.0 statistical software. Measurement data were expressed as mean ± standard deviation, and GraphPad Prism 9.0 was used for graphing. The statistical significance of differences between the two groups was determined using Student's *t*-test. One-way ANOVA followed by the SNK method was used for comparison among multiple groups. Statistical significance was set at *P* value < 0.05.

### Supplementary Information

Supplementary Information.

## Data Availability

All data are contained in the article and the Supplementary Materials.
