# Author Correction: On the mechanism of wogonin against acute monocytic leukemia using network pharmacology and experimental validation

**DOI:** 10.1038/s41598-024-65501-7

**Published:** 2024-06-24

**Authors:** Xixi Wang, Yanfei Wang, Jing Chen, Qinyao Wang, Zhongjian Liu, Yijie Yin, Tonghua Yang, Tao Shen, Yalian Sa

**Affiliations:** 1 Center for Clinical Medicine Research, The First People’s Hospital of Yunnan Province (Affiliated Hospital of Kunming University of Science and Technology), Kunming, 650032 China; 2 Medical School, Kunming University of Science and Technology, Kunming, 650500 China; 3 Department of Hematology, The First People’s Hospital of Yunnan Province, Kunming, 650032 China; 4 Department of Respiratory and Critical Care Medicine, The First People’s Hospital of Yunnan Province (Affiliated Hospital of Kunming University of Science and Technology), Kunming, 650032 China

Correction to: *Scientific Reports* 10.1038/s41598-024-60859-0, published online 02 May 2024

In the original version of this Article, Affiliation 2 was incorrect.

‘Medical school, Kunming University of Science and Technology, Kunming 650032, China.’

now reads,

‘Medical School, Kunming University of Science and Technology, Kunming 650500, China.’

In addition, Yalian Sa was affiliated only with ‘Medical school, Kunming University of Science and Technology, Kunming 650500, China.’

The correct Affiliations are listed below:

- ‘Center for Clinical Medicine Research, The First People’s Hospital of Yunnan Province (Affiliated Hospital of Kunming University of Science and Technology), Kunming 650032, China.’
- ‘Medical School, Kunming University of Science and Technology, Kunming 650500, China.’

The original Article has been corrected.

