# Supplementary material for: On the mechanism of wogonin against acute monocytic leukemia using network pharmacology and experimental validation
Source: Sci Rep. 2024 May 2;14:10114. doi: 10.1038/s41598-024-60859-0 (PMC11065882; doi:10.1038/s41598-024-60859-0)
Supplement: Supplementary file 1 — Supplementary Information. [file 41598_2024_60859_MOESM1_ESM.pdf]

Original Image for Fig 10A AKT.
Exposure 1:

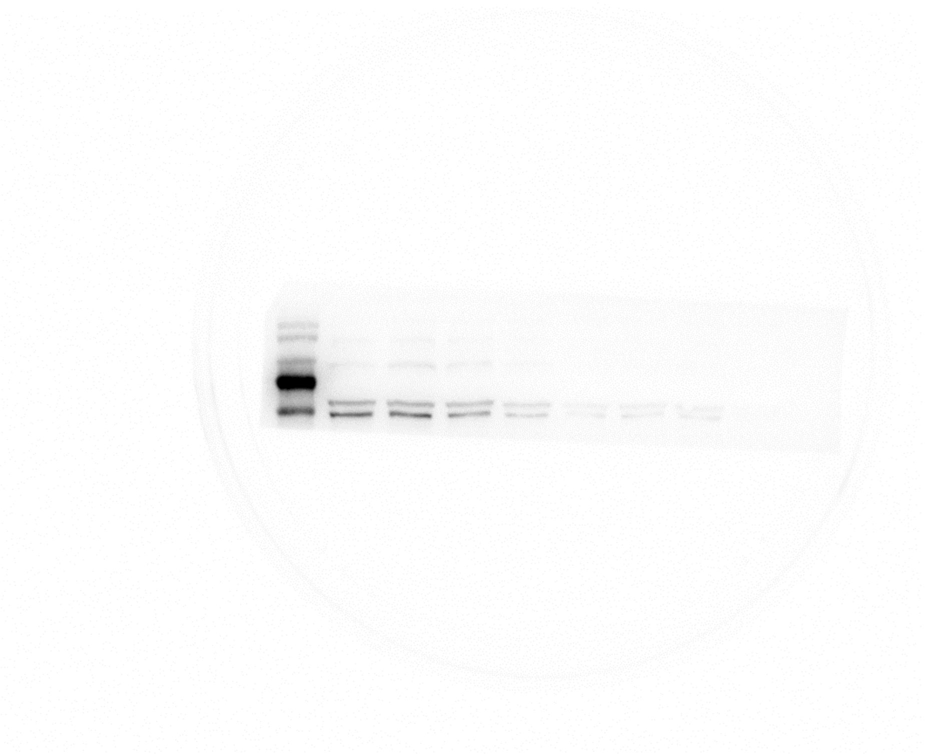

Exposure 2:

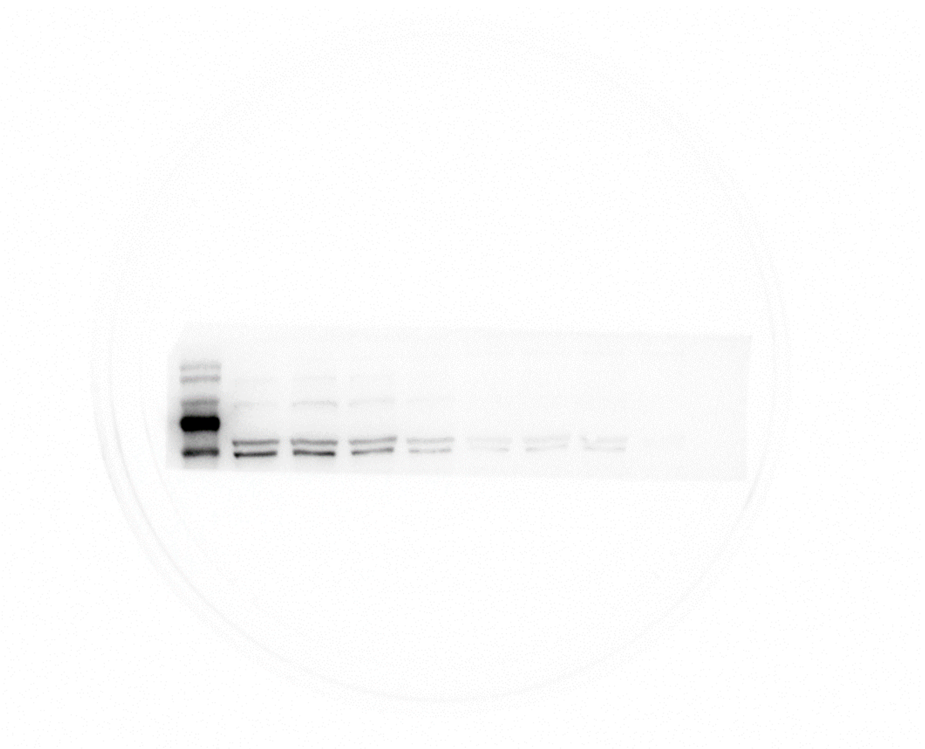

Exposure 3:

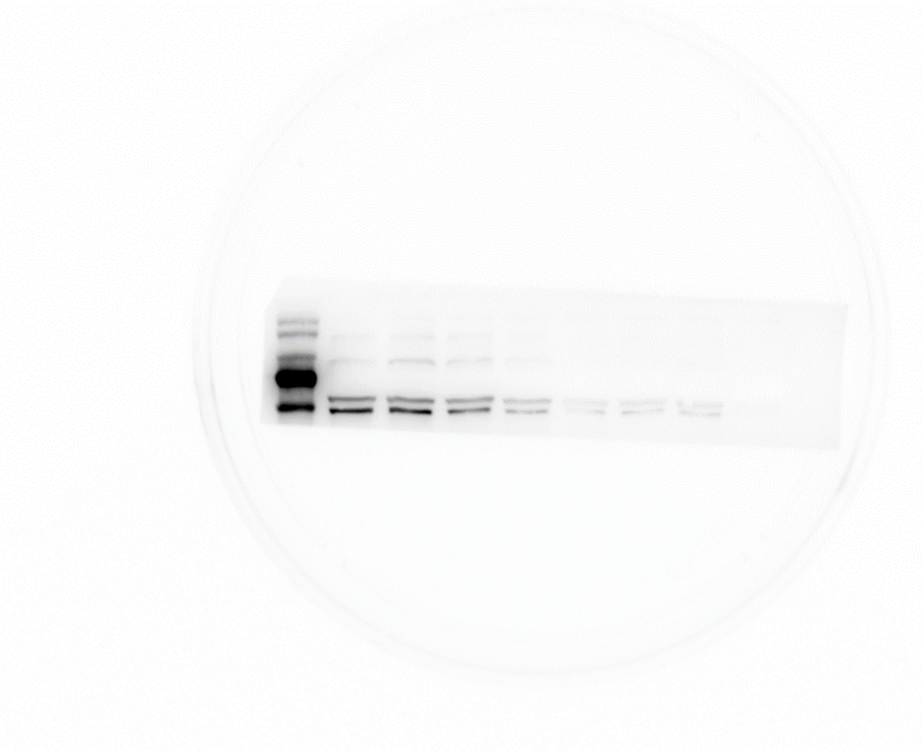

Exposure 4:

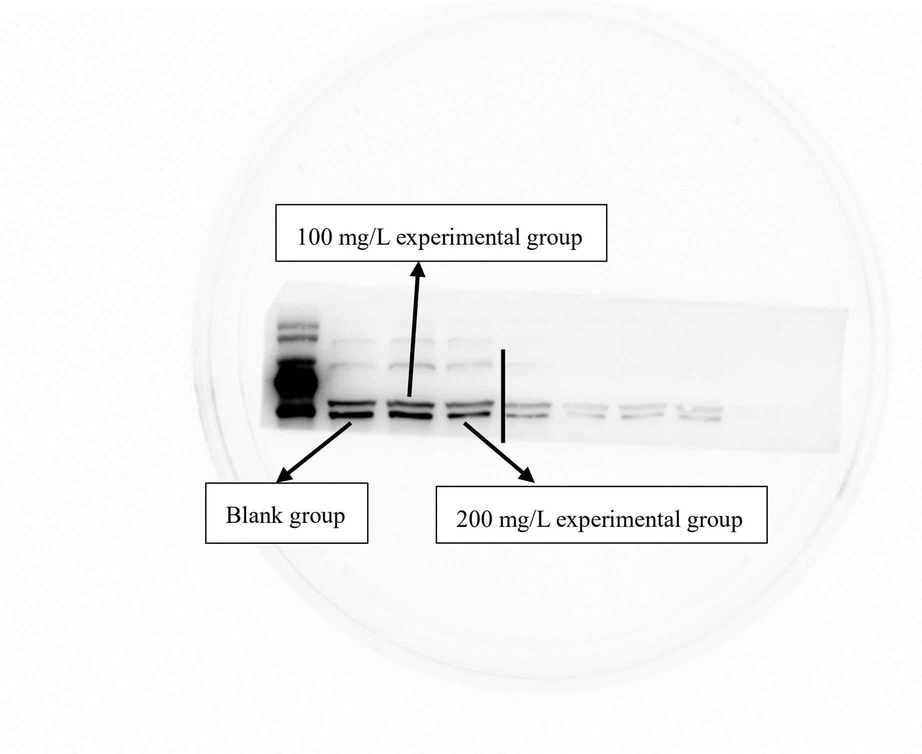

The original blot detects the protein expression of AKT. The first three lanes counted from left to right are the groups for this experiment: the blank group, the 100 mg/L experimental group, and the 200 mg/L experimental group.

Original Image for Fig 10A p-AKT.
Exposure 1:

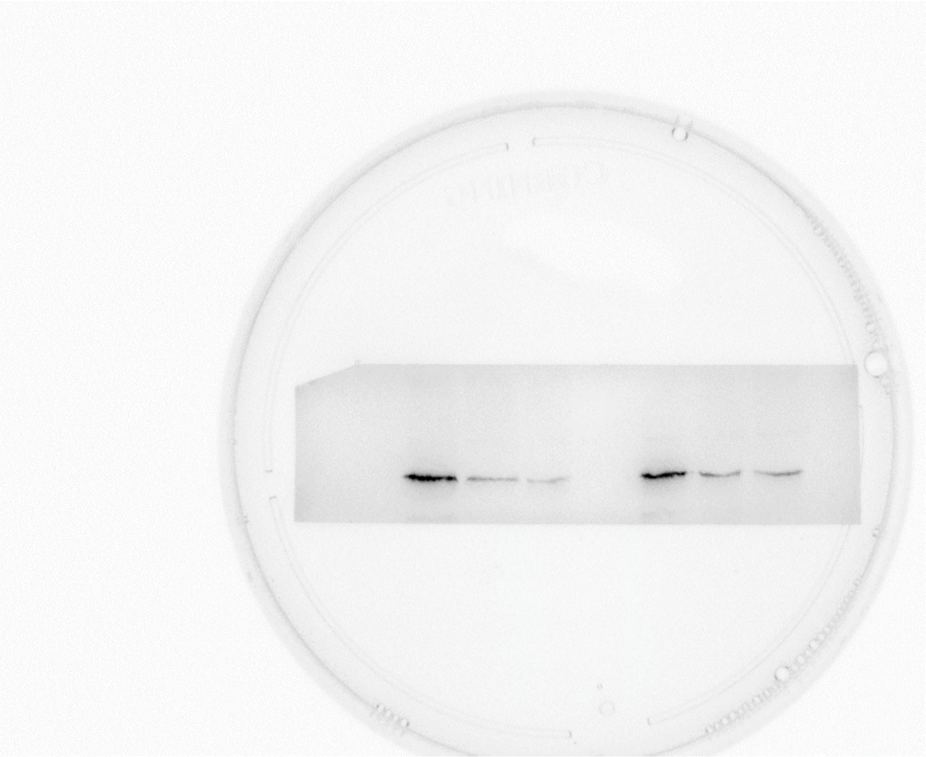

Exposure 2:

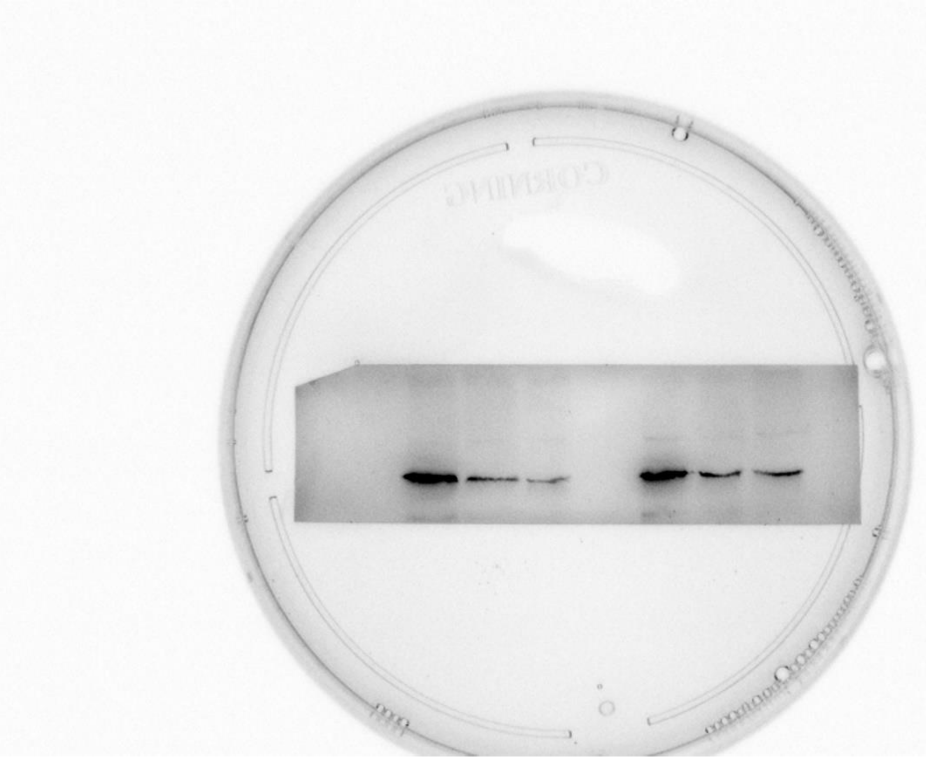

Exposure 3:

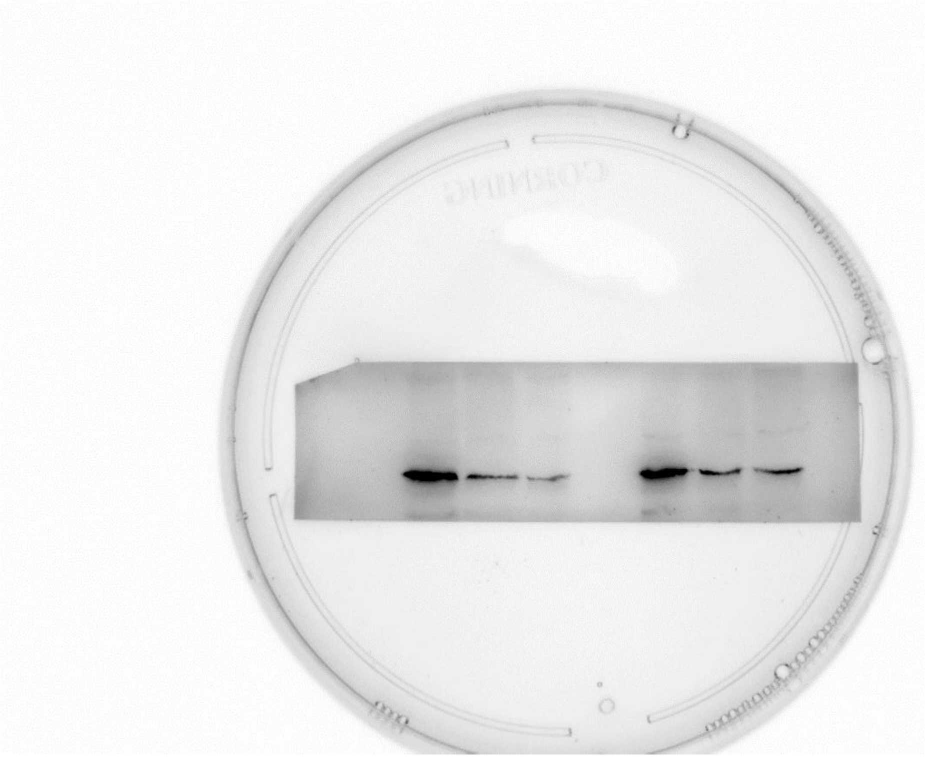

Exposure 4:

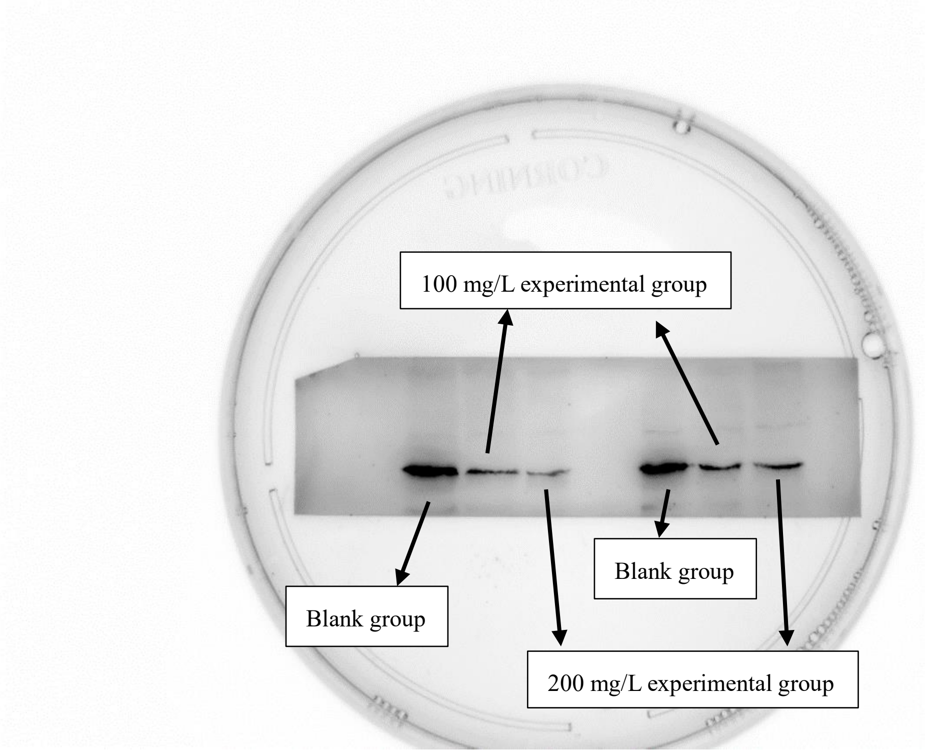

The original blot detects the protein expression of p-AKT. The first three lanes counted from left to right are the groups for this experiment: the blank group, the 100 mg/L experimental group, and the 200 mg/L experimental group. The last three lanes are duplicate experiments with the same grouping as the previous ones.

Original Image for Fig 10A GAPDH.
Exposure 1:

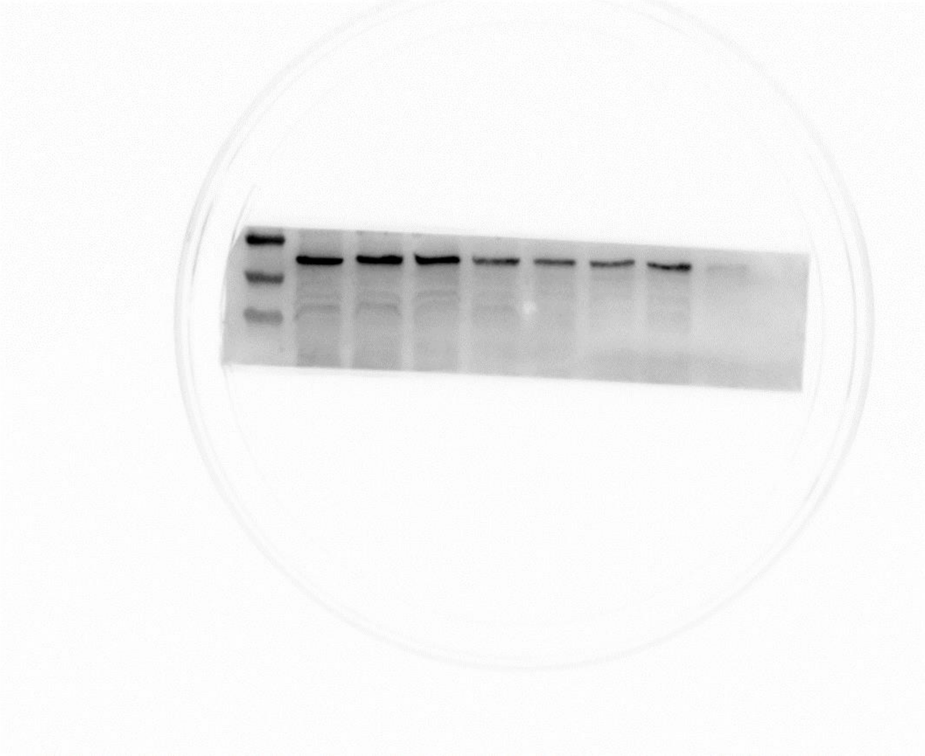

Exposure 2:

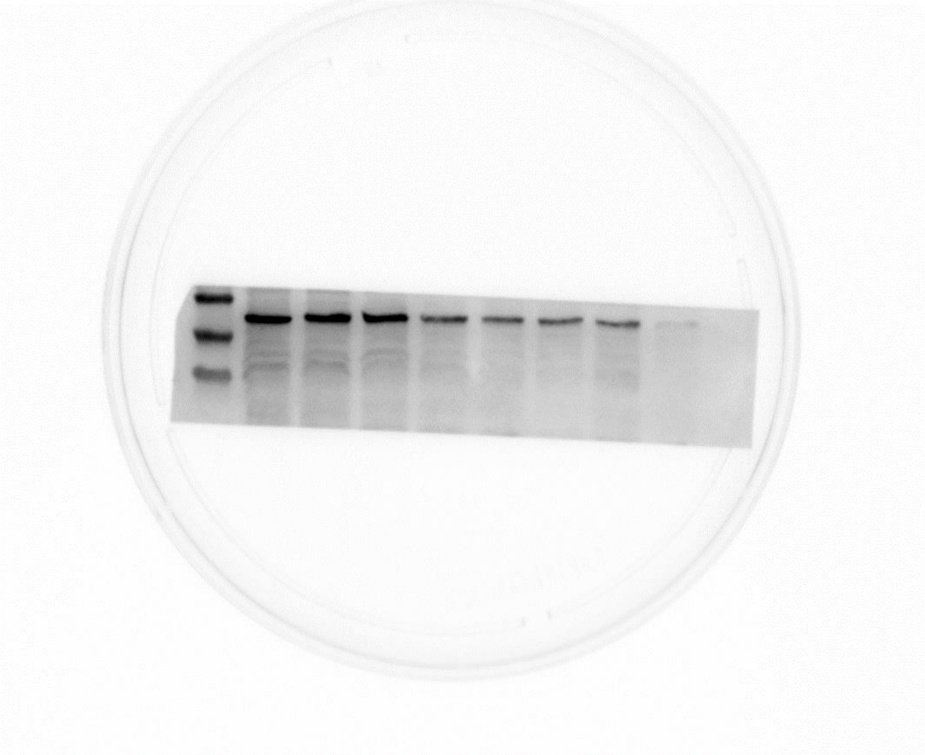

Exposure 3:

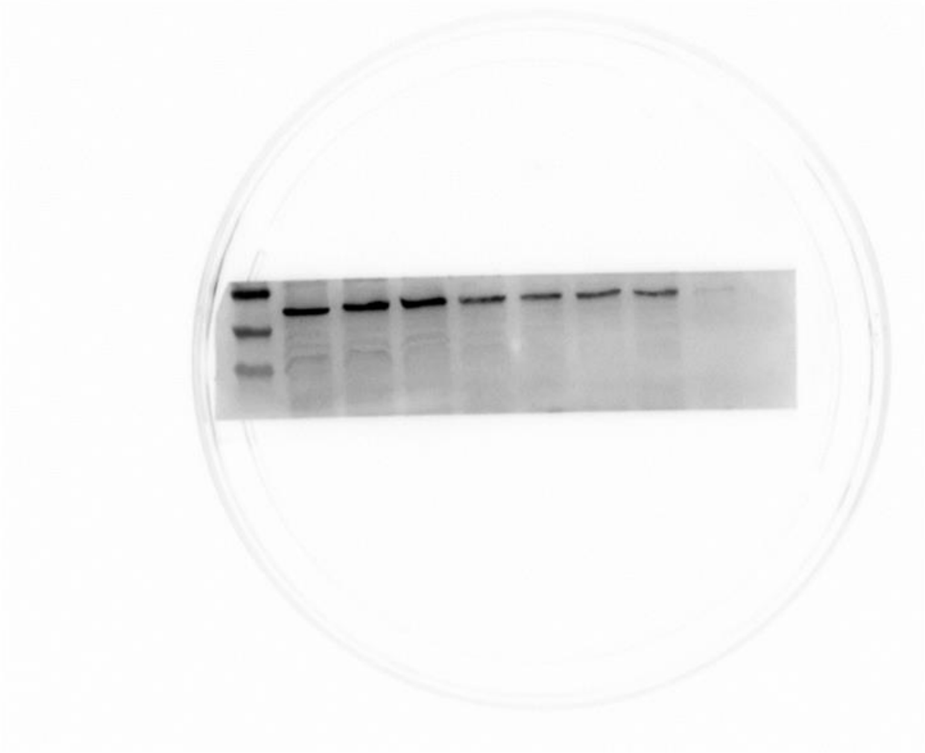

Exposure 4:

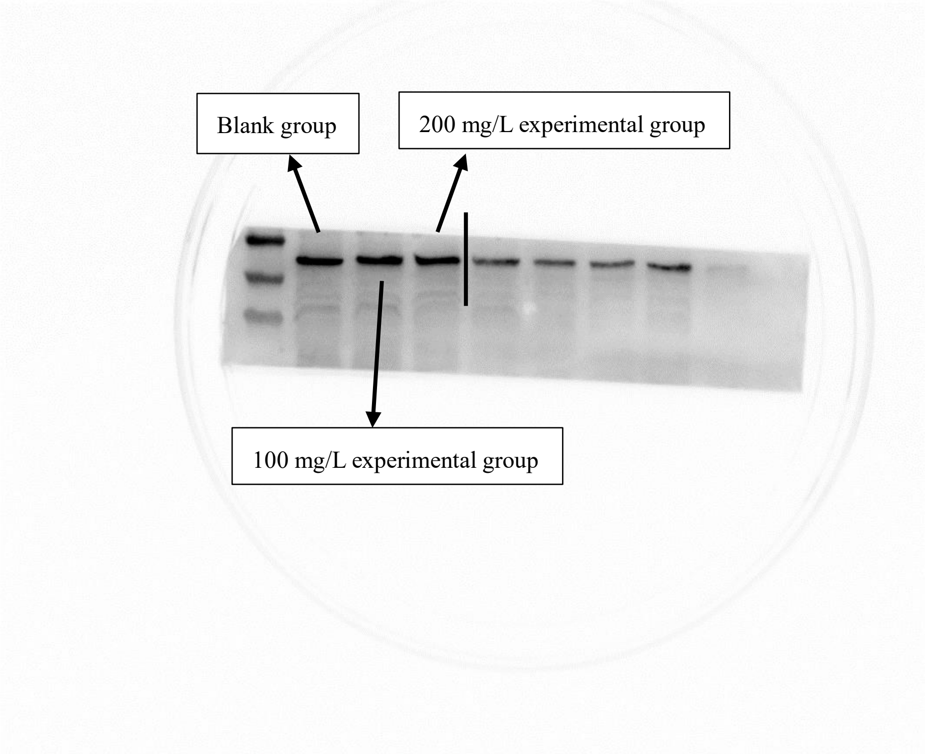

The original blot detects the protein expression of GAPDH. The first three lanes counted from left to right are the groups for this experiment: the blank group, the 100 mg/L experimental group, and the 200 mg/L experimental group
